# Evaluation of on-farm indicators of gilt reproductive performance potential at 21 days of age^1^

**DOI:** 10.1093/tas/txaa210

**Published:** 2020-11-20

**Authors:** Kayla M Mills, Allan P Schinckel, Jebadiah G Stevens, Theresa M Casey, Kara R Stewart

**Affiliations:** 1 Department of Animal Sciences, Purdue University, West Lafayette, IN; 2 Ag Production Enterprises, Greensburg, IN

**Keywords:** gilt, reproduction, vulva size

## Abstract

Selection of replacements for the sow herd is one of the most important facets in swine production. Although our current methods of selection are effective, there is still a large amount of variation in sow reproductive performance traits such as pigs per sow per year (PSY). Therefore, the objective of this study was to determine if on-farm phenotypic traits at 21 d postnatal (PN) or perinatal environmental factors could predict sow reproductive performance. Data were prospectively collected from 2,146 gilts born on a commercial sow production facility and included birth and weaning weights, vulva length and width at 21 d PN, birth and nursing litter size, days nursed, average daily gain from birth to weaning, and age at first estrus. Of the initial animals, 400 (17%) were selected for the sow herd, 353 remained after removal of animals culled for non-reproductive reasons. Animals were assigned to 1 of 5 reproductive performance categories based on observation of estrus or pigs per sow per year (PSY) across two farrowings: High Fertility (HF; 23%; *n* = 82; ≥26 PSY), Middle Fertility (MF2; 12%; *n* = 43; 20–25 PSY), Low Fertility (MF3; 15%; *n* = 54; <20 PSY), Infertile-Estrus (IFe; 10%; *n* = 36; estrus, no pregnancy), and Infertile-No Estrus (IFno; 39%; *n* = 138; no estrus, no pregnancy). Generalized linear model analysis indicated vulva width (*P* = 0.03) was related to PSY, however, it only explained 1.5% of the total variation in PSY. To determine if preweaning variables were predictive of gilt fertility outcome, animals were grouped as those that became pregnant (*n* = 179) or not (*n* = 174). Vulva width tended to be greater in fertile animals versus infertile (*P* = 0.07). Binomial regression analysis revealed a positive relationship between vulva width and gilt fertility, however, this relationship is not strong enough to make sow herd selection decisions.

## INTRODUCTION

A gilt's reproductive efficiency has a major effect on economic profitability in the swine industry. A common method to measure reproductive efficiency is pigs weaned per female per year (PSY) (Stalder et al., 2003, 2019; Stalder, 2009). The 2019 average of PSY was 26.08 for farms in PigCHAMP database and 26.61 in MetaFarms (MetaFarms, 2020; PigCHAMP, 2020). Another way to measure sow farm reproductive efficiency is sow replacement rate. Despite high yielding sows, the removal rates of animals from sow herds averaged 45% across the US in 2018, with culling primarily due to poor reproductive performance. In addition to a high sow removal rate, 38.5–51.1% of gilts selected as potential replacements are culled due to reproductive failure (Roongsitthichai et al., 2013; Li et al., 2018).

A negative relationship exists between the litter size in which a gilt is raised in and her lifetime reproductive efficiency (Robison, 1981; Van der Steen, 1985; Bartol et al., 2006; Bagnell et al., 2009; Bartol et al., 2009), and is likely due to animals in smaller litters having heavier birth weights which is positively related to colostrum consumption (Le Dividich et al., 2005; Morton et al., 2019). Gilts that consume greater amounts of colostrum tend to be heavier at birth and gain more weight postnatally (de Passille and Rushen, 1989; Milligan et al., 2002), as well as display signs of estrus earlier and have better lactation performance as sows than their low-colostrum counterparts (Vallet et al., 2015). Together demonstrating that low colostrum consumption is associated with impaired reproductive performance in sows (George et al., 2019).

Because of the strong relationship between a gilt's reproductive potential and pre-weaning nutrition and growth, swine operations have implemented perinatal piglet care and fostering programs that minimize competition among littermates and maximize access to suckling. These measures often result in standardization of nursing litter size, so animals have similar growth rates and size at weaning. At weaning, farm managers are tasked with identifying gilts to be reared for replacement breeding females instead of entering the food chain. However, the minimization of differences in size and growth rates of animals at weaning makes selection of females with the greatest reproductive potential challenging (Robison, 1981; Van der Steen, 1985; Serenius and Stalder, 2006; Bagnell et al., 2009; Bartol et al., 2009).

To maximize economic returns, there is a need to identify animals with the greatest reproductive potential prior to their entering the breeding herd. An efficient gilt management system has three selection timepoints: in the nursery, start of boar exposure, and when gilts enter puberty (Patterson et al., 2010). Average age at first estrus, which marks puberty, is the most predictive parameter of sow reproductive efficiency (Patterson et al., 2010). However, average age of estrus is 240 d and at this point of production the producer has already invested in an animal that may fail to ever cycle. Vulva width by 95–115 days of age has been associated with a gilt's ability to achieve puberty by 200 days of age (Graves et al., 2020), and vulva width at 105 days was found associated with sow productivity through two parities (Romoser et al., 2020). The objective of this prospective longitudinal study of over 2,000 gilts born on a commercial sow production facility was to determine if there was a relationship of preweaning traits such as average daily gain from birth to weaning, birth and weaning weight, and vulva size at weaning with reproductive efficiency and longevity in sow breeding herd.

## MATERIALS AND METHODS

### Animals

**Figure 1.**
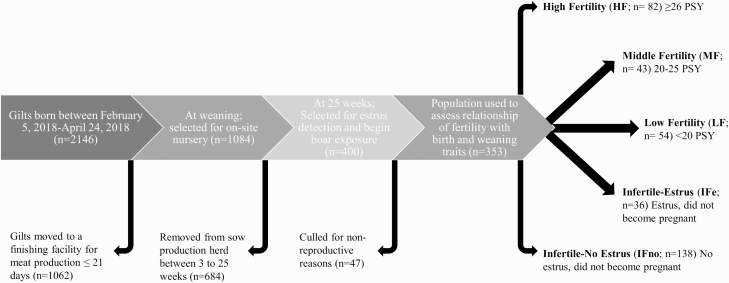
Timeline of the study. Timeline of the study representing different timepoints of selection and different fertility phenotypes. PSY, Pigs per sow per year.

All procedures involving animals were reviewed and approved by the Institutional Animal Care and Use Committee (1605001416). Gilts (*n* = 2,146) born between 5 February 2018 and 24 April 2018, on a commercial farm in central Indiana were enrolled in the longitudinal observational study (Figure 1). Animals at the commercial facility were maternal lines PIC 1070 (Large White x L019) and C29 (Landrace x PIC 1070) which are specifically bred for preweaning piglet performance and sow lifetime productivity. Between postnatal d 2 and 3 piglets were individually identified by an ear tag and weighed and processed (200 mg iron, tail docking, Baytril, Tylan 50) as per routine management on the farm. Following individual identification, litter sizes were standardized to 14 ± 1 piglets by farm technicians through cross-fostering between litters of similar aged piglets. Litter (farrowing date, birth litter size, number weaned) and individual piglet (birth weight, weaning weight) information were entered into MetaFarms (MetaFarms, Inc., Burnsville, MN) database for each sow.

**Figure 2.**
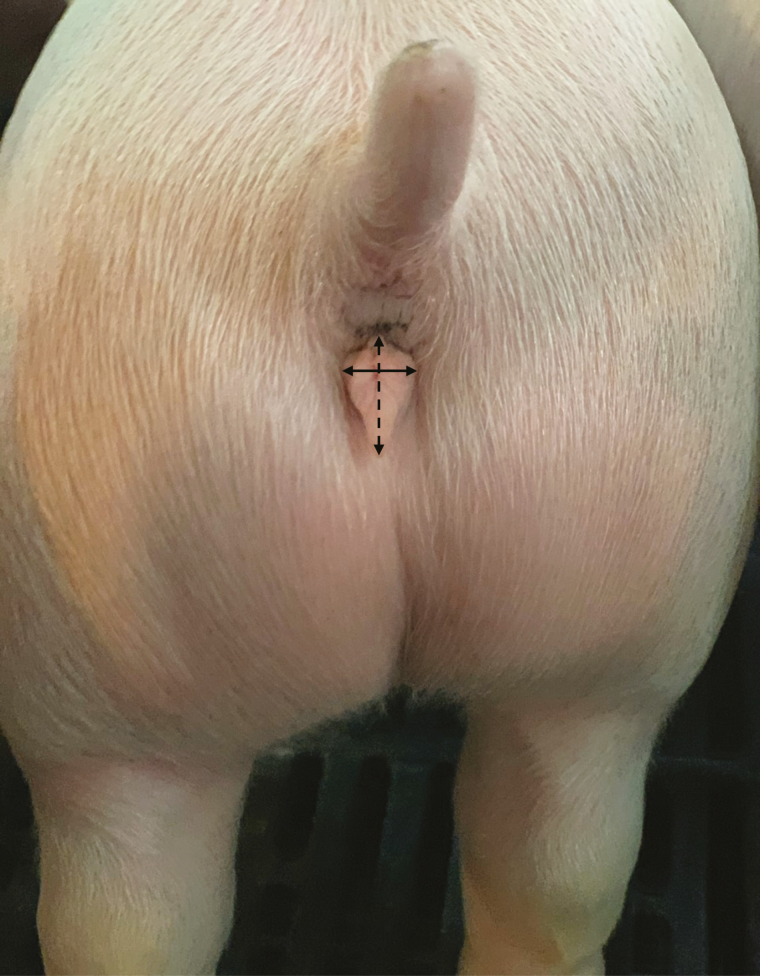
Vulva morphometric measurement. Vulva length (dashed line) and width (solid line) were measured at 21 ± 4 d using digital calipers from the very top of the vulva to the bottom of the vulva and at the widest part of the vulva, respectively. Vulva measurements were taken in millimeters. The gilt pictured is 21 days of age.

Piglets were weaned at 21 ± 4 d, and 1,084 gilts from the initial pool of animals were selected as replacements for the farm's onsite nursery (Figure 1). Animals not selected as replacement gilts were transferred to an offsite wean to finish facility for market production. At the time of weaning gilts in the on-site nursery were weighed and vulva lengths and widths were measured using digital calipers (Fisherbrand Traceable Digital Carbon Fiber Calipers, Fisher Scientific Company L.L.C., Pittsburgh, PA). Vulva length was measured from the bottom most point of the vulva to the top of the vulva (Figure 2), and width was measured at the widest part of the vulva (Figure 2). Vulva metrics were entered and stored in a research database.

At 25 weeks of age, gilts were moved from the nursery into the onsite gilt development unit (GDU). Gilts in the GDU received daily, full-contact boar exposure to induce puberty and screen for signs of estrus. Gilts were observed for signs of estrus daily beginning at 25 weeks of age. Date of gilt's first and second estrus were recorded. When a second estrus was detected, gilts were moved to a gestation crate and bred using artificial insemination (AI) on their third estrus. If gilts did not show any signs of estrus following three weeks of heat detection they were given a full dose of P.G. 600 (Intervet America, Inc., Millsboro, DE) to induce estrus and were bred on the subsequent heat. Gilts that did not respond to P.G. 600 were culled from the selection pool. Data on reproductive history to include day of first estrus, breeding date, and treatment with P.G. 600 were entered into Metafarms database.

### Categorization of Fertility Groups

**Figure 3.**
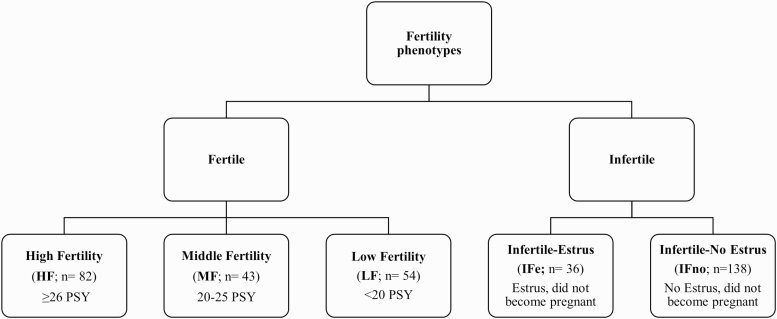
Dendrogram of fertility phenotype categories. The population used to assess the relationship of fertility with birth and weaning traits was divided into two main classes: Fertile or Infertile. Within those classifications, fertile animals were divided into 3 subclasses based on pigs per sow per year (PSY): High Fertility (HF), Middle Fertility (MF), and Low Fertility (LF). Infertile animals were divided into two subclasses based on whether they exhibited estrus: Infertile-Estrus (IFe) and Infertile-No Estrus (IFno).

On September 22, 2019 performance data from breeding herd animals that had birth weights, weaning weights, and vulva measurements recorded (*n* = 400) were extracted from Metafarms reports which included date of birth, date of first boar exposure, dates of estrus detection, date of mating, herd removal (cull) date, reason for removal, farrowing date and number of piglets born alive. Date of data extraction was selected to allow time for at least two farrowings of all study animals from time of birth. Animals that were culled from the breeding herd for non-reproductive reasons such as lameness, disease, or leg injuries, were removed from data set. The 353 that remained were divided into two main classes based on whether an animal was fertile (Figure 3). If animals were fertile, they were divided into three subclasses based on PSY. High Fertility (HF; *n* = 82) animals were defined as sows that had at least 26 PSY. Middle Fertility (MF; *n* = 43) gilts were characterized as sows that had 20–25 PSY. Sows that had less than 20 PSY were classified as Low Fertility (LF; *n* = 54). PSY was chosen as it is an index that encompasses multiple facets of fertility and was calculated by totaling the number of piglets weaned from a sow during her first productive year up to two parities. Infertile animals were divided into two subclasses based on whether they exhibited estrus. Infertile-Estrus (IFe; *n* = 36) were gilts that showed signs of estrus but did not become pregnant. Gilts that did not show any signs of estrus following boar exposure and P.G. 600 were characterized as Infertile-No Estrus (IFe; *n* = 138).

### Statistical Analysis

Analysis of production variables by fertility category was performed using the GLM procedure of SAS 9.4 (SAS Inst. Inc., Cary, NC). All other analyses were completed in R (v 3.5.1). A generalized linear model was used to assess whether birth weight, weaning weight, vulva length, vulva width, birth litter size, days nursed, nursing litter size, average daily gain from birth to weaning, and age at first estrus contributed to the variation in PSY. Predictors of sow reproductive performance were assessed using binomial regression analyses where:

$$\begin{array}{ll} Probability_{Bred}=(21\;d\;of\;age\;vulva\;width,\;mm) & \times 0.126091.14445 \end{array}$$

A value of *P* < 0.05 was used to determine significance and values of 0.05 ≤ *P* ≤ 0.10 were considered trends.

## RESULTS

Of the 400 that entered the breeding herd with birth and weaning weight data, 82 (23%) were considered highly fertile animals (HF; ≥26 PSY), and 138 (39%) categorized as infertile (IFno) due to failure to show any signs of estrus upon boar exposure by 29 weeks of age, and failure to respond to hormonal induction of estrus. Intermediate phenotypes were characterized as sows that had 20–25 PSY (MF; 12%; *n* = 43), sows that had less than 20 PSY (LF; 15%; *n* = 54), and gilts that showed signs of estrus during boar exposure but did not become pregnant (IFe; 10%; *n* = 36; Figure 3). Gilts that were not selected for the gilt development unit from the initial replacement selection pool (*n* = 684) had lower birth and weaning weights, smaller vulva widths, lower average daily gain (ADG) from birth to weaning, and had more piglets in their birth litter than gilts selected for the final breeding herd at 25 weeks of age (Table 1; *P* < 0.01).

**Table 1.** Preweaning characteristics of gilts that were culled at 25 weeks of age or selected for the final breeding herd from the initial replacement weaning pool (*n* = 1,084)*

Preweaning traits	Culled from initial selection pool^†^ (*n* = 684)	Gilts selected for final breeding herd (*n* = 400)	SE	*P*-value
Birth weight, kg	1.698	1.775	0.019	<0.001
Weaning weight, kg	4.979	5.738	0.080	<0.001
Vulva length, mm	13.48	13.03	0.195	0.124
Vulva width, mm	8.810	9.077	0.089	0.010
Birth litter size	13.87	12.99	0.125	<0.001
Days nursed	21.52	21.37	0.110	0.276
Nursing litter size	13.60	13.52	0.045	0.112
ADG^**^ from birth to weaning	0.152	0.186	0.004	<0.001

*This table compares the 684 animals culled between weaning and 25 weeks of age to the 400 gilts selected at 25 weeks of age for breeding.

^**^Average daily gain.

^†^Gilts selected at weaning as potential replacements.

Vulva width (Table 2; *P* = 0.03) and nursing litter size (Table 2; *P* = 0.05), were differentiating factors in PSY. However, all factors included in the statistical model only accounted for 3.4% of the total variation in PSY. A reduced statistical model that only included vulva width and nursing litter size was used to further evaluate whether vulva width or nursing litter size influenced PSY. Vulva width was found to be the only differentiating factor (reduced model A; Table 2; *P* = 0.04) which suggests that nursing litter size was slightly correlated to a variable that was removed from the full model. Therefore, a second reduced model that included only vulva width (reduced model B) was used to determine how much variation in PSY was explained by vulva width. Vulva width was found to explain 1.5% of the variation in PSY and was still a differentiating factor (Table 2; *P* = 0.03). When all phenotypes were compared, vulva width was not different (Table 3; *P* = 0.35). There were no differences among all phenotypes for any of the other pre-weaning variables from the full model (Table 3).

**Table 2.** Independent variables of statistical models and their influence on the variation in PSY*

Statistical model	Independent variables	Sum of squares	*P-*value
Full model**	Birth weight, kg	4.64	0.84
	Weaning weight, kg	10.8	0.76
	Vulva length, mm	22.0	0.66
	Vulva width, mm	590	0.03
	Birth litter size	32.7	0.60
	Days nursed	382	0.07
	Nursing litter size	447	0.05
	ADG^†^ from birth to weaning	12.6	0.74
	Age at first estrus	338	0.09
Reduced model A^‡^	Vulva width, mm	633	0.04
	Nursing litter size	200	0.26
Reduced model B^§^	Vulva width, mm	805	0.03

*Three generalized linear models were used to determine if on-farm factors influenced sow reproductive performance defined in this study as pigs weaned per sow per year (PSY). The Full Model included all measured on-farm traits. Predictors from the Full Model that were significant or approached significance were included in Reduced Model A. Reduced Model B assessed the influence of the only significant predictor on the variation observed in PSY.

**Adjusted *r*^2^ = 0.034.

^†^Average daily gain.

^‡^Adjusted *r*^2^ = 0.012.

^**§**^Adjusted *r*^2^ = 0.015.

**Table 3.** On-farm characteristic means among all phenotypes and their differentiating phenotype*

Parameters	HF^**^	MF^†^	LF^‡^	IFe^§^	IFno^||^	SE	*P-*value
Birth weight, kg	1.81	1.81	1.73	1.79	1.75	0.07	0.64
Weaning weight, kg	5.95	5.68	5.88	5.78	5.53	0.24	0.43
Vulva length, mm	12.9	13.2	13.4	13.1	13.2	0.42	0.72
Vulva width, mm	9.31	9.30	9.08	8.74	8.95	0.30	0.35
Birth litter size	13.1	13.0	12.9	13.2	13.0	0.44	0.99
Days nursed	21.5	21.7	21.2	21.1	21.3	0.38	0.51
Nursing litter size	13.6	13.7	13.4	13.7	13.4	0.13	0.23
ADG^$^ from birth to weaning	0.19	0.12	0.20	0.19	0.18	0.01	0.39
Age at first estrus, days	186	186	186	189	–	1.40	0.42

*This table highlights means for each on-farm characteristic included in the Full Model for all fertility phenotypes.

^**^High fertility; ≥26 PSY.

^†^Middle fertility; 20–25 PSY.

^‡^Low fertility; <20 PSY.

^§^Infertile-estrus; estrus, did not become pregnant.

^||^Infertile-no estrus; No Estrus, did not become pregnant.

^$^Average daily gain.

**Figure 4.**
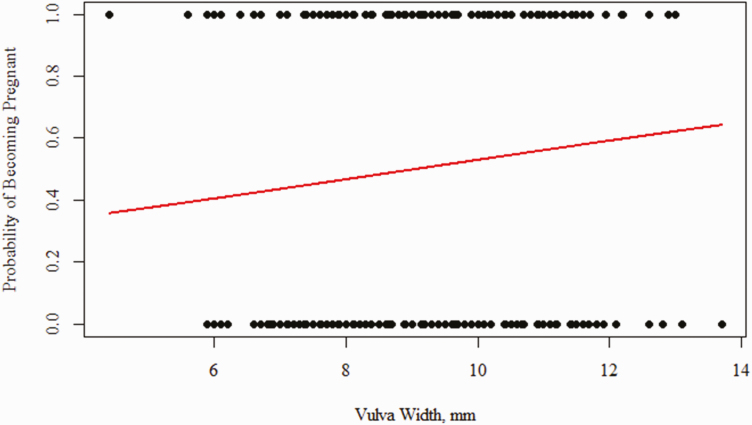
Vulva width as a predictor of gilt fertility at 21 ± 4 days of age. Binomial regression analysis was used to determine if vulva width at 21 ± 4 days of age could be a predictor of a gilt fertility. A Nagelkerke *r*^2^ was calculated to determine if there was a relationship between vulva width at 21 ± 4 days of age and gilt fertility.

**Figure 5.**
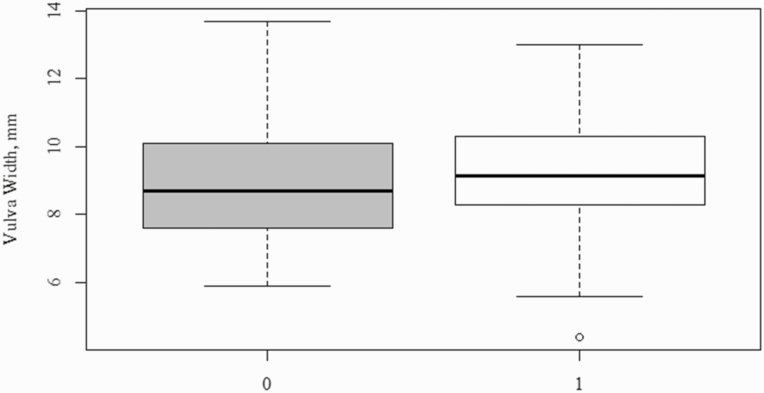
Distribution of vulva width between infertile (0) and fertile gilts (1). The figure depicts the distribution of vulva width at 21 ± 4 days of age in both infertile and fertile gilts.

To determine if preweaning variables were predictive of fertility outcome, animals were grouped as those that were fertile (*n* = 179) or infertile (*n* = 174). Vulva width tended to be greater in animals who were fertile (Table 4; Figure 5; *P* = 0.07). Fertile animals also tended to come into estrus 3 days earlier than open animals (Table 4; *P* = 0.10). Binomial regression analysis revealed a positive relationship between vulva width and probability of becoming pregnant later in life (Figure 3), however, the relationship is not strong (Nagelkerke *r*^2^ = 0.014) due to the variation in vulva width among phenotypes (Figure 4). Binomial regression analysis was also completed using age at first estrus, birth weight, and weaning weight as predictors of pregnancy, however, these parameters were not predictive of pregnancy status.

**Table 4.** Fertile vs. infertile on-farm characteristic means and *P*-values*

On-farm characteristic	Fertile, *n* = 179	Infertile, *n* = 174	SE	*P*-value
Birth weight, kg	1.79	1.76	0.05	0.53
Weaning weight, kg	5.87	5.61	0.14	0.14
Vulva length, mm	13.1	13.2	0.20	0.89
Vulva width, mm	9.23	8.89	0.15	0.07
Birth litter size	13.0	13.1	0.21	0.77
Days nursed	21.5	21.2	0.13	0.27
Nursing litter size	13.6	13.5	0.09	0.45
ADG^**^ from birth to weaning	0.19	0.18	0.01	0.26
Age at first estrus, days	186	189	1.13	0.10

*This table highlights the comparison of on-farm characteristics between fertile and infertile gilts.

^**^Average daily gain.

## DISCUSSION

Achieving an adequate return on investment in replacement gilts is a challenge that the swine industry currently faces. The leading cause of removals from sow herd is related to poor reproductive performance. This is due in part to the lack of the ability of the producer to identify reproductively sound gilts early in life (MetaFarms, 2020; PigCHAMP, 2020). Because the perinatal environment is associated with long-term fertility, we aimed to determine whether several easily measured morphological and developmental markers could be related to fertility outcomes. We found that among all traits assessed, only vulva width at weaning was weakly related to long-term fertility of gilts.

In this study, consistent with modern swine farm management, we found the combination of genetics, cross-fostering, weaning schedule, and selection of gilts into the gilt development unit (GDU) resulted in the similar birth weights, nursing litter sizes, weaning weights, average daily gain from birth to weaning, number of days nursed, and birth litter size across all fertility phenotype groups. The very light birthweight piglets were not selected into the on-site nursery which could explain the high degree of similarity for animals that made it into the GDU who had an average birth weight of 1.72 kg. However, it is interesting to note that animals culled between weaning and 25 weeks of age were born into a larger litter, had a smaller body size at both birth and weaning, and a lower ADG between birth and weaning. All of these traits are indicators that animals culled between weaning and 25 weeks likely to consumed less colostrum, thus negatively affecting the overall developmental trajectory of the animal (de Passille and Rushen, 1989; Milligan et al., 2002; Le Dividich et al., 2005; Devillers et al., 2011; Quesnel et al., 2012; Morton et al., 2019).

During the first two weeks of life, the gilt's reproductive tract undergoes morphologic and molecular changes across the uterus and cervix (Bartol et al., 2006) and into the vagina (Harlow et al., 2019b). The amount of colostrum consumed in the first days postpartum is related to long-term fertility (Vallet et al., 2015), and affects reproductive development centrally and peripherally (George et al., 2019). Morphologic and molecular changes in the gilt's upper and lower reproductive tract are affected by colostrum intake (Bagnell et al., 2009; Bartol et al., 2009; Harlow et al., 2019a), with several studies linking relaxin in milk to postnatal reproductive tract development in swine (Bagnell et al., 2005; Frankshun et al., 2012).

The vulva is an external extension of the female reproductive tract, and previous studies have shown a relationship between sow reproductive efficiency to vulva width at 95 days of age (Graves et al., 2020) likely correlated to pubertal increases in estrogen. Our findings revealed that there is a positive relationship between vulva width at weaning and the probability of a gilt becoming pregnant. Fertile gilts also had numerically heavier weaning weights than the infertile gilts which suggests they may have had a subtle advantage in the perinatal environment. The association between colostrum intake and reproductive tract development before weaning, suggests that vulva size at weaning may be an indicator of colostrum consumption and therefore reproductive status later in life. Due to the physical similarities at 21 days of age, it is difficult to discern which animals will become the most prolific gilts on the farm. Future research will thus likely need to investigate the potential in evaluating the molecular environment of the gilt's reproductive tract at 21 days of age to determine if biomarkers can be used to differentiate between phenotypes.

Identifying gilts with the greatest reproductive potential at 21 days of age rather than at 175 days of age would be incredibly valuable to the swine industry. Our data indicated that there is a positive relationship between vulva width and probability of a gilt becoming pregnant, however, this relationship was not very strong due to the variation in vulva size at weaning and thus cannot be used alone when making gilt selection decisions. Vulva width may be more beneficial when used in conjunction with current selection strategies.
